# Machine learning hypothesis-generation for patient stratification and target discovery in rare disease: our experience with Open Science in ALS

**DOI:** 10.3389/fncom.2023.1199736

**Published:** 2024-01-04

**Authors:** Joseph Geraci, Ravi Bhargava, Bessi Qorri, Paul Leonchyk, Douglas Cook, Moses Cook, Fanny Sie, Luca Pani

**Affiliations:** ^1^NetraMark Corp, Toronto, ON, Canada; ^2^Department of Pathology and Molecular Medicine, Queen's University, Kingston, ON, Canada; ^3^Centre for Biotechnology and Genomic Medicine, Medical College of Georgia, Augusta University, Augusta, GA, United States; ^4^Arthur C. Clarke Center for Human Imagination, School of Physical Sciences, University of California San Diego, San Diego, CA, United States; ^5^Department of Biomedical and Molecular Science, Queens University, Kingston, ON, Canada; ^6^Science and Research, Roche Integrated Informatics, F. Hoffmann La-Roche, Toronto, ON, Canada; ^7^Department of Surgery, Queen's University, Kingston, ON, Canada; ^8^Department of Medical Biophysics, University of Toronto, Toronto, ON, Canada; ^9^Department of Psychiatry and Behavioral Sciences, Leonard M. Miller School of Medicine, University of Miami, Coral Gables, FL, United States; ^10^Department of Biomedical, Metabolic, and Neural Sciences, University of Modena and Reggio Emilia, Modena, Italy

**Keywords:** augmented intelligence, Open Science, targeted therapy, combination therapy, collaboration, machine learning, artificial intelligence, ALS

## Abstract

**Introduction:**

Advances in machine learning (ML) methodologies, combined with multidisciplinary collaborations across biological and physical sciences, has the potential to propel drug discovery and development. Open Science fosters this collaboration by releasing datasets and methods into the public space; however, further education and widespread acceptance and adoption of Open Science approaches are necessary to tackle the plethora of known disease states.

**Motivation:**

In addition to providing much needed insights into potential therapeutic protein targets, we also aim to demonstrate that small patient datasets have the potential to provide insights that usually require many samples (>5,000). There are many such datasets available and novel advancements in ML can provide valuable insights from these patient datasets.

**Problem statement:**

Using a public dataset made available by patient advocacy group AnswerALS and a multidisciplinary Open Science approach with a systems biology augmented ML technology, we aim to validate previously reported drug targets in ALS and provide novel insights about ALS subpopulations and potential drug targets using a unique combination of ML methods and graph theory.

**Methodology:**

We use NetraAI to generate hypotheses about specific patient subpopulations, which were then refined and validated through a combination of ML techniques, systems biology methods, and expert input.

**Results:**

We extracted 8 target classes, each comprising of several genes that shed light into ALS pathophysiology and represent new avenues for treatment. These target classes are broadly categorized as inflammation, epigenetic, heat shock, neuromuscular junction, autophagy, apoptosis, axonal transport, and excitotoxicity. These findings are not mutually exclusive, and instead represent a systematic view of ALS pathophysiology. Based on these findings, we suggest that simultaneous targeting of ALS has the potential to mitigate ALS progression, with the plausibility of maintaining and sustaining an improved quality of life (QoL) for ALS patients. Even further, we identified subpopulations based on disease onset.

**Conclusion:**

In the spirit of Open Science, this work aims to bridge the knowledge gap in ALS pathophysiology to aid in diagnostic, prognostic, and therapeutic strategies and pave the way for the development of personalized treatments tailored to the individual’s needs.

## Introduction

The convergence of artificial intelligence (AI), machine learning (ML), and data science is adding new dimensions to the advancement of our understanding of disease biology (Yang, n.d.). Traditional drug discovery and development is a high-risk, time- and cost-consuming process that takes, on average, over a decade and over $1 billion for each new drug approved for clinical use (Schaduangrat et al., 2020; Sun et al., 2022). By leveraging advanced AI/ML computational methods, meaningful insights can be derived from existing biological data (Iskar et al., 2012). As a result, pharmaceutical and biotechnology companies are beginning to incorporate these approaches to drive innovation in drug discovery (McKinsey, n.d.).

Given this paradigm shift, there is an urgent need to evolve infrastructure to foster the intersection between domain experts in AI and data science with life sciences (McKinsey, n.d.). As Judea Pearl once noted, “…data are profoundly dumb.,” suggesting that mathematics and computer science need to come together to develop methods that can extract valuable insights from data that are reflected in the causal factors driving the phenomenon being modeled while engaging biologists to provide contextual and plausibility insights (Pearl and Mackenzie, 2018). Technological efforts inspired by this mission are reported in this paper.

Currently, approximately 30% of the world’s data volume is generated from the healthcare industry (RBCCM, 2018). This estimation is only going to get higher as AI/ML techniques and our expertise of extracting insights evolves at a phenomenal pace (Dash et al., 2019; Hirschler, n.d.). There may be several barriers associated with accessing and extracting meaningful insights from healthcare data, including patient privacy and data integrity, but these roadblocks are actively being addressed by fostering collaborations with the ML community while embracing Open Science approaches to tackle healthcare challenges (Dash et al., 2019; Seh et al., 2020; Batko and Ślęzak, 2022; Miguel Cruz et al., 2022; Singhal and Carlton, n.d.). At its core, Open Science encourages transparency and collaboration with all stakeholders throughout the scientific research cycle, from conception and design to data production, analysis, and dissemination (OECD, 2015). The benefits of Open Science are well documented, and it is crucial that researchers are properly equipped with the knowledge and skills required to navigate an Open Science landscape (Zečević et al., 2020). It has become evident that Open Science will play an essential role in addressing health inequity, improving patient engagement, and treatment access for all patients (Holzmeyer, 2019; Norori et al., 2021). However, this requires increasing awareness of the power of Open Science and a collaborative effort to reduce the barriers that will enable better engagement in Open Science activities. Here, we demonstrate the value of Open Science to produce useful insights into amyotrophic lateral sclerosis (ALS) through partnerships between an AI/ML startup and academic collaborators.

ALS, also known as motor neuron disease or Lou Gehrig’s disease, is a relentlessly progressive neurodegenerative and neuromuscular disease that results in the loss of motor neurons that control voluntary muscles (Johns Hopkins, n.d.). ALS is the most common motor neuron disease in adults and the third most common neurodegenerative disease after Alzheimer’s disease and Parkinson’s disease (Logroscino et al., 2018). Worldwide, ALS incidence is estimated to be 1.9 per 100,000 people per year, while the prevalence of ALS at any given time is estimated to be about 4.5 per 100,000 people (Barceló et al., 2021; Park et al., 2022). Most concerning, the number of ALS cases worldwide is projected to increase by 69% from 2015 to 2040 to approximately 376,000 cases a year, primarily due to the aging of the world’s population, especially in developing countries (Arthur et al., 2016).

Over 90% of ALS cases are thought to be sporadic, with the remaining 10% accounting for familial ALS (Nowicka et al., 2019). Many environmental and genetic risk factors are thought to contribute to sporadic ALS; however, none have been clearly linked to ALS onset (Nowicka et al., 2019). ALS is known to be a complex genetic disease, with a liability threshold model for ALS proposing that cellular damage accumulates over time due to genetic factors present at birth and exposure to environmental risks throughout life (Simpson and Al-Chalabi, 2006). The disease can exhibit as either bulbar or limb onset, with the former associated with accelerated disease course and a poorer prognosis, necessitating swift and robust therapeutic response. In contrast, the more gradual progression observed in limb onset affords a larger window for deliberating potential treatment approaches (Masrori and Van Damme, 2020). Due to notable disease heterogeneity, the diagnosis, progression, and prognosis vary for each individual, with early symptoms including stiff muscles, muscle twitches, gradual increasing weakness, and muscle wasting. The disease eventually advances to the point where most individuals lose critical motor function, ultimately resulting in paralysis and early death, usually from respiratory failure (Goutman et al., 2022). There is currently no cure for ALS, and treatment is focused on improving symptoms (Nowicka et al., 2019).

Disease heterogeneity, late-stage recruitment into pharmaceutical trials, and inclusion of phenotypically admixed patient cohorts are some of the key barriers to successful clinical trials. In this new era of open science, ML approaches and large international datasets offer unprecedented opportunities to appraise candidate diagnostic, monitoring, and prognostic markers (Grollemund et al., 2019; Ziff et al. 2023).

In this paper, we aim to expand on previously reported work to demonstrate the potential for using modern ML technologies to learn from the many smaller datasets that are publicly available (Pun et al., 2022). Smaller datasets are typically considered unsuitable for ML, but with the continuing advancement of ML and the utility of large language models (LLMs) to amplify signals from small datasets, work which demonstrates that pertinent insights are possible from smaller datasets is important. Here, we utilize an Open Science approach, taking advantage of a public ALS dataset from the ALS Kaggle challenge, with no further integration of other data.

In the context of ALS research within the Kaggle challenge and using a shared dataset, various groups undertook analytical investigations to pinpoint key variables linked to different ALS pathologies. Notably, one group identified robust activation of p53 in *TARDBP* and sporadic ALS subgroups, while its activity was still elevated but considerably diminished in FUS and SOD1 mutant ALS cases (Ziff et al., 2023). Another group used RefMap to identify ALS risk genes, integrating genome-wide association study (GWAS) data with molecular profiling to reveal genes associated with ALS-related molecular phenotypes like TDP-43 mislocalization, hypoexcitability, and disruptions in neurotrophic signaling. Furthermore, this study identified *ADAMTSL1, BNC2, KANK1,* and *VAV2* as significantly enriched rare variants linked to ALS, with correlations to disease severity (Zhang et al., 2022). A separate investigation identified variants in 22 genes associated with sporadic ALS patients, including *NDUFS4, AC106707.1, ZC3H7B, AC023095.1*, and *CCD59*, among others. Markedly, *NDUFS4*, similar to SOD1, plays a role in antioxidant defense mechanisms and stands out as a gene of interest in ALS research. Notably, this latter group successfully identified a set of genetic markers capable of detecting ALS in >30% of patients with a 99% confidence interval (Logan et al., 2022). Finally, the PandaOmics study identified high-confidence therapeutic targets from iPSC-differentiated motor neurons (diMN)-derived and CNS data (Pun et al., 2022).

Using this same dataset, we set out to expand upon the drug target list provided in that work, and to report on targets that overlapped with their analysis, as further validation using an ML “playground” environment, NetraAI (Qorri et al., 2020; Choi et al., 2021; Cook et al., 2023), that allows biological content experts to interact with ML-generated hypotheses to evaluate the findings for context and plausibility. Further, we present evidence that this is a well-defined subclass of bulbar initiated ALS patients whose genetic underpinnings corroborate the axonal transport machinery that is currently considered a likely etiological component for ALS pathophysiology. We provide novel insights that support this theory that can play an important role for future therapeutics. This Open Science approach aims to bridge the gap between advanced ML techniques and human medical expertise through AI. Our goal was to use these techniques to provide a synopsis of potential drug targets for ALS.

## Methodology

### Datasets

Answer ALS is the largest collaborative effort in ALS bringing together multiple research organizations and key opinion leaders. Over 800 ALS patients and 100 healthy controls from 8 neuromuscular clinics distributed across the United States were enrolled in this project. A blood sample was collected at the first visit of each participant and iPSC lines were generated from peripheral blood mononuclear cells extracted from whole blood via an episomal iPSC reprogramming system. The consortium generated multi-omics data comprising of genomic, epigenomic, transcriptomic, proteomic, laboratory test, medical records, and other data (Baxi et al., 2022). We used transcriptomic records within the files named bulbar_vs_limb.csv and ctrl_vs_cas.csv which are currently being expanded for future research and competitions. These files were available on Kaggle for academia and industry. The former data file is meant to differentiate between how ALS initializes, specifically in the bulbar region or limbs, allowing our system to extract key sets of genes that are active in different patient subpopulations. The latter data file was used to differentiate biological mechanisms that play a role in ALS in general, and to generate genetic hypotheses about ALS subpopulations. The data used in the preparation of this article were obtained from the Answer ALS Data Portal (AALS-01184). For up-to-date information on the study and access to the data please visit https://www.answerals.org/.

### Analysis

An ML playground environment called NetraAI (Qorri et al., 2020; Choi et al., 2021; Cook et al., 2023) was made available to scientists at the Gladstone Institute. This allowed medical experts to interact with the ML-generated hypotheses to evaluate the findings and examine the etiological factors that were being suggested. Here, we bridged the gap that exists between advanced ML techniques and human medical expertise through augmented intelligence (Crigger et al., 2022). The methods used for the generation of the hypotheses that led to the target classes described in this paper consisted of ML methods paired with systems biology methods. In this context, we refer to ML-generated hypotheses as proposed insights about a patient subpopulation that satisfy the following criteria:

- The insight must be about a specific subset of samples that the AI finds and include a multi-factor signature that pertains to this subpopulation.
- The insight must pass significance testing by comparing the precisely defined subpopulation against other collections of samples or patients.
- The insight is further strengthened by being passed through a LLM in order to shape it according to the existing literature and to transform it into a human readable statement.

An important issue is the small number of samples within the dataset used, as we did not augment our process with other data such as literature or other genetic datasets. Our process is based on authentic limitations that exist in rare disease clinical trials, which begins with inherently small sample sizes of patients. For this reason, we built an ML pipeline using methods suitable for smaller sample sizes. By allowing the algorithms to segment the patient samples into clusters of varying confidence, and extracting precisely what factors are driving each cluster, we have a set of hypotheses that can be tested statistically and by human ALS experts. Smaller datasets do not have the sample size to accurately represent the variety of manifestations of ALS, but the sample we had access to did provide insights into statistically significant patient subpopulations. The novelty of our approach stems from the following insights:

- Small datasets need to be partitioned into *explainable and unexplainable subsets*.
- The explainable subsets are hypotheses, which are sets of variables and collections of samples that pass statistical significance testing. The unexplainable subsets are groups of patients that represent unknowns with respect to predictions from the resulting models. In other words, this process infuses the resulting models with the ability to be clear about what subtypes of patients it can make reliable predictions about, and those that will require more data and future efforts.
- Knowledge of these explainable subsets and their driving variables improve leave out cross validation statistics significantly.

These subpopulations were then used to extract features that were supported through significance testing and expert validation. These were then used to seed biological network analyses and hypothesis generation. This is an example of augmented intelligence, where ML methods are used to enhance human expertise, especially when datasets are limited in sample size. This process was implemented as follows:

1. Each dataset had a column with labels as it pertains to control subjects versus ALS patients, or limb versus bulbar initiation.
2. Due to the smaller sample sizes of the datasets, we utilized Random Forest, Gradient Boosted Trees, support vector machines, UMAP, and methods previously described, to partition the data into subpopulations (Qorri et al., 2020; Choi et al., 2021; Cook et al., 2023). The sequence of these methods allows one to extract a set of genes that acted coherently to define different patient classes. Each of these sets of genes along with a subset of patients/subjects will now be referred to as a hypothesis, as defined above.
3. The genes implicated for each hypothesis are then entered into a systems biology platform. The systems biology platform utilizes data on how proteins interact and co-express. These data are derived from Warde-Farley et al. (2010) and utilized in the following way:
  - Each gene implicated by the methods outlined (Qorri et al., 2020; Choi et al., 2021; Cook et al., 2023) has a graph grown around it according to adjustable parameters. The genes that come from the hypotheses are considered *parent nodes* and the number of *daughter node*s to be included is a parameter, e.g., *maximum degree*. Another parameter is the number of connections allowed for each daughter node (i.e., *maximum daughter degree*).
  - Graphs are grown according to protein interaction, gene co-expression, gene interactions, or domain similarity, and any of these in any combination can be selected. If an interaction exists between any two proteins/genes, according to one of these parameters, an edge is formed between the pairs. The edges can be weighted based on a metric derived from publications about the interaction and reflects the confidence in that interaction.
  - Network centrality measures such as eigenvector, betweenness, and closeness centrality measures are used to derive a score for each gene in the network (Geraci et al., 2012; Sekhar and Ambedkar, 2020). A linear combination of node metrics was used to determine which nodes were the most important from a drug target perspective. The parent nodes derived from the ML methods applied to the patient population dataset are used to evaluate the graph distance to other nodes implicated by the interaction data. Nodes that are farther away are penalized than those that are closer. However, the methods consider that high-degree nodes can be lethal, as drugging them could disrupt multiple critical molecular pathways. By using a linear combination of node metrics, one can utilize a combination of scores to capture different aspects of these graph theoretic metrics as outlined previously (Galan-Vasquez and Perez-Rueda, 2021; Viacava Follis, 2021). For instance, even though how many connections a protein has is important, targeting high degree proteins can also cause toxicity. This should be balanced with proteins that have the potential to modulate disease despite not being high degree but being connected to proteins that are. Thus, by combining multiple scores one can consider different molecular influencers that act through different topological mechanisms (Galan-Vasquez and Perez-Rueda, 2021; Viacava Follis, 2021).
  - Potential targets are ranked according to their ability to interfere with a process that aligns with the ML-derived hypotheses, as described. Ideally, the parameters of the process are chosen so that lethal targets are avoided as well as ineffective proteins, which are far from the parent nodes. This is done by ranking all resulting daughter nodes by distance, degree, and centrality measures.
  - Targets are also linked with pathways and potential binding chemical compounds if they exist.
4. The results of these computations, including the ranking of potential drug targets, the pathways they belong to, and binding chemical compounds were the outputs of the algorithms used. These outputs were used to decide which targets to include.

**Figure 1:**
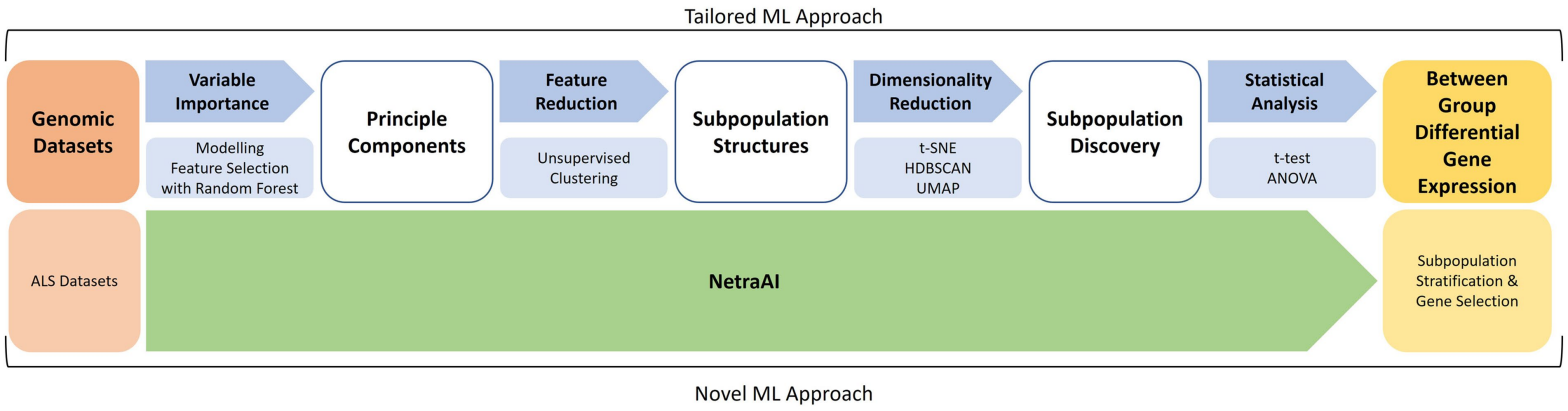
Machine learning approach for patient subpopulation and gene set discovery. Using two ALS datasets, a tailored ML approach consisted of feature selection with random forest, unsupervised clustering, cluster exploration with t-SNE, HDBSCAN, UMAP, and statistical analyses to obtain between-group differential gene expression for subpopulations of ALS patients. These were used to extract hypotheses about driving genes and then used to seed the previously described biological network analyses.

The ML methodology utilized is outlined in Figure 1 and has previously described in more detail (Qorri et al., 2020; Choi et al., 2021; Cook et al., 2023). This was the methodology used to segment the patient population before applying the biological network methods described above.

### Analytical methods and parameter choices

For a foundational understanding of the data’s structures and to facilitate feature reduction, a series of methods and parameters were adopted. During data preprocessing, features were centered by subtracting their respective means. Recognizing the varied feature scales, the data underwent standardization to ensure every feature converged to a mean of 0 with a standard variance of 1. When implementing the PCA, we opted for the “full” solver, a choice influenced by the manageable size of our dataset which promised a thorough decomposition. To zero in on the optimal components, a significant focus was placed on the cumulative explained variance, ensuring our emphasis was on principal components accounting for 95% of the total variance. This approach was further cross-referenced by inspecting the “elbow” of the scree plot. The significance of features was gauged through their loading values, where features with pronounced absolute values were considered for the selection process. Furthermore, these features from PCA loadings were assessed against our domain expertise. This ensured that the pruned feature set was not just technically sound but also contextually relevant, particularly in the lens of potential ALS drug targets. Before embarking on these steps, multicollinearity among features was scrutinized using the variance inflation factor (VIF). Features breaching a VIF of 10 were given a closer look. With the dataset’s size being on the smaller side, outliers posed a risk of disproportionate influence. To counteract this, data distributions were visually examined and complemented with statistical methods geared toward outlier identification and assessment.

Our study also made use of the Random Forest method, an ensemble learning technique used for its capabilities in both classification and regression tasks. By leveraging a collection of decision trees, each being trained on a randomized assortment of data subsets and features, an aggregate predictive outcome was pursued. The primary intent here was the validation of features unearthed using our unique techniques. The dataset was strategically bifurcated, earmarking 80% for training purposes and the remaining for testing. Stratified sampling was integral in this division, a necessity arising from the class imbalance observed in our target variable. Utilizing the Scikit-learn library available in Python, we initialized the Random Forest with parameters such as 500 trees, the criterion set as “gini,” max depth restricted to 30, min_samples_split and min_samples_leaf defined as 5 and 2, respectively, and finally, a consistent random_state of 42. Post training, the Gini importance was extracted, which subsequently played a pivotal role in ranking features. A predetermined threshold was set at 0.005 for feature importance, selecting only those that surpassed this benchmark. Their inclusion was further bolstered by an out-of-bag (OOB) error measuring 0.03. For evaluations, a fresh Random Forest model was trained using the cherry-picked features, which was then validated against the testing subset. Finally, grid search was utilized specifically for hyperparameter fine-tuning, resulting in optimal parameters of n_estimators at 550 and max_depth solidified at 32.

In our approach with t-SNE, we settled on settings such as a perplexity of 30. This was largely due to its alignment with smaller datasets, effortlessly balancing between local and global structures. Accompanying parameters included a learning rate of 200, capped iterations at 1000, early exaggeration of 12, a balancing angle of 0.5, and a swift PCA-based initialization for the sake of faster convergence. Additionally, the metric was strictly defined as “euclidean.” We chose “exact” for the method parameter, offering an advantage over the Barnes-Hut approximation for petite datasets, all while minimizing complexity.

HDBSCAN clustering was configured as follows: The Minimum Cluster Size was fixed at 5, with the Minimum Samples mirroring this value by default. The Cluster Selection Method was distinctly marked as “eom” or Excess of Mass. In this phase, the Allow Single Cluster option was purposefully deactivated. Alpha was precisely set at 1.0, keeping avenues open to experiment with elevated values. The metric was once again aligned with the previous selection of “euclidean,” and Core Distance was singularly set at 1 to bolster computation times.

Lastly UMAP was used to decipher the intricate interrelations among patients, echoing discoveries from our in-house methods. Crucial parameters here were the n_neighbors fixed at 15, min_dist tailored to 0.1, the metric used was “euclidean,” a spread adjusted to 1.0, and the min_dist_fraction set at 0.1 for this study.

This comprehensive approach, underlined by these carefully chosen parameters, was our roadmap to robust, interpretable results, all the while side-stepping pitfalls like overfitting and computational lags.

### Target confidence evaluation

TargetMine, an Open Source and peer reviewed tool that uses known genetic relationships with disease, biological pathway data, and current drug information was used to provide confidence levels for the targets we discovered with our NetraAI system (Chen et al., 2022). We compiled a comprehensive list of genes, all of which are included in this study. This list was formatted into a comma-separated value (CSV) file for computational analysis. The dataset was uploaded to the TargetMine platform, where we specifically selected *Homo sapiens* as the reference organism. In the “Analyse Data” tab, we initiated the analysis procedure, where it was imperative to rectify the nomenclature of several genes to ensure system recognition. Following the successful recognition of all the genes, we proceeded with the detailed analysis. TargetMine generated a downloadable report, of which the disease pathway enrichment section was of particular interest to our study. This section provided the statistical significance measures that underpinned our findings and facilitated the stratification of our target genes based on confidence levels and putative functionalities. All these data including pathway enrichment provide the data to derive significance values for the targets discovered by our process.

## Results

### ALS drug targets replicated by NetraAI

Several studies have attempted to identify key players in ALS pathology with hopes of elucidating relevant drug targets for this fatal disease (Batra et al., 2019; Hedl et al., 2019; Nowicka et al., 2019; Wu et al., 2019). However, many identified targets relate to mitochondrial dysfunction, protein aggregation, RNA processing, axonal transport, oxidative stress, apoptosis, *SOD1,* phosphorylation, and the neuromuscular junction (Batra et al., 2019). Most methods to extract these targets are based on symptoms and the mechanisms of disease development and progression; however, due to the heterogeneity of the disease, it is important to identify key players that can be druggable in specific subsets of ALS patients. Several ML approaches have identified key genetic targets, and using NetraAI, we were able to verify several of the same gene targets that have been recently reported (Table 1) as well, we identified several genes that belong to the same gene family as those previously reported (Table 2; Pun et al., 2022). The functions reported in Tables 1, 2 are based on the protein family function as well as supporting literature that discusses a proposed mechanism or function. Within Table 1, *DNM3TA*, *ERN1*, *HSPD1, PPIA, VCP, MAP3K5, MAKPK1, NOS1, PTK2, PTPRC,* and *RARA* were previously identified high-confidence therapeutic targets from iPSC-differentiated motor neurons (diMN)-derived and CNS data that belonged to the druggable classes defined by PandaOmics, with supportive evidence on their ALS or neurodegeneration, and ranked as the top-50 targets in at least one of the meta-analyses (Pun et al., 2022). In contrast, *PPP3CB*, was identified as a novel therapeutic target in the previous reported findings (Pun et al., 2022). The findings presented in Table 2 represent gene targets that belong to the same protein family as other targets identified by the PandaOmics study.

**Table 1:** Previously found drug targets by PandaOmics replicated by our methodology.

Drug target	Function	References
*DNMT3A* (DNA methyltransferase 3 alpha)	DNA methylation; apoptosis	Wong et al. (2013)
*ERN1* (Endoplasmic Reticulum to Nucleus Signaling 1)	Sensor for endoplasmic reticulum unfolded protein response (UPR); protein aggregation, apoptosis	Medinas et al. (2017) and Montibeller and de Belleroche (2018)
*HSPD1* (heat shock protein family D member 1)	Innate immune response; FUS pathology, inflammation	Gorter et al. (2019)
*PPIA* (Peptidylprolyl Isomerase A)	Survival and growth pathways; mediator of inflammation; TDP-43 pathology	Pasetto et al. (2017)
*VCP* (valosin containing protein)	Protein segregation and degradation; DNA repair and replication; cell cycle regulation; mitochondrial dysfunction	Koppers et al. (2012) and Scarian et al. (2022)
*PPP3CB* (protein phosphatase 3 catalytic subunit beta)	Calmodulin-binding activity; protein phosphatase 2B binding activity; NFAT signaling cascade; apoptosis; cellular degradation; protein aggregate degradation	RGD (n.d.)
*MAP3K5* (Mitogen-Activated Protein Kinase Kinase Kinase 5)	Mediator of apoptosis signaling	Medinas et al. (2017)
*MAPK1* (Mitogen-Activated Protein Kinase 1)		Pasetto et al. (2017), Gorter et al. (2019)
*NOS1* (Nitric oxide synthase 1)	Multifunctional signaling molecule and neurotransmitter; associated with *SOD1* upregulation (role in ALS)	Almer et al. (1999), Kiernan et al. (2011), and Tripathi et al. (2020)
*PTK2* (Protein Tyrosine Kinase 2)	Cell adhesion, migration, and survival; ubiquitous proteasome system and protein degradation	Lee et al. (2020)
*PTPRC* (Protein Tyrosine Phosphatase Receptor Type C)	Transmembrane receptor protein phosphatase activity; immune cell function	Galan-Vasquez and Perez-Rueda (2021)
*RARA* (Retinoic acid receptor alpha)	Autophagy	Viacava Follis (2021)

Genes listed in this table are the same as those reported in the PandaOmics study (Pun et al., 2022).

**Table 2:** Targets belonging to the same protein family identified by our methodology.

Drug target	Function	References
*NR3C2* (Nuclear Receptor Subfamily 3 Group C Member 2)	Aldosterone signaling pathway	Yu et al. (2022) and Ziff et al. (2022)
*KCNB1* (Potassium Voltage-Gated) Channel Subfamily B Member 1	Regulator of cortex and hippocampus neuronal firing	Yu et al. (2016)
*P2RY12* (purinergic receptor)	Neuroinflammation; platelet aggregation	Amadio et al. (2014), Volonté et al. (2016), and Morillas et al. (2021)
*SCYL3* (SCY1 Like Pseudokinase 3)	Neuronal function and survival; cell migration and adhesion	Pelletier (2016) and Kuliyev et al. (2018)
*SLC25* (Solute Carrier Family 25 Member 4, 5, 6, 18, 22)	Mitochondrial function; neuron energy production Apoptosis Mitochondrial function L-glutamate transmembrane transport; aspartate transmembrane support; malate–aspartate shuttle Mitochondrial glutamate transporter	GeneCards-SLC25A18 (n.d.), Häggmark et al. (2014), Kuliyev et al. (2018), and Aykaç and Şehirli (2020)
*RPS6KA1* (ribosomal protein S6 kinase A1)	Mediator of cell survival; cell growth, motility, survival, and proliferation	UniProt, (n.d.) and Iridoy et al. (2019)
*RPS6KA2* (ribosomal protein S6 kinase A2)		GeneCards-RPS6KA2 (n.d.) and Zhang (2023)
*KCNV2* (potassium voltage-gated channel modifier subfamily V member 2)	Neurotransmitter release regulation; neuronal excitability	Wu et al. (2006) and Gore et al. (2010)

Genes listed in this table belong to the same protein family as those reported in the PandaOmics study (Pun et al., 2022).

### Novel ALS targets uncovered by NetraAI

**Figure 2:**
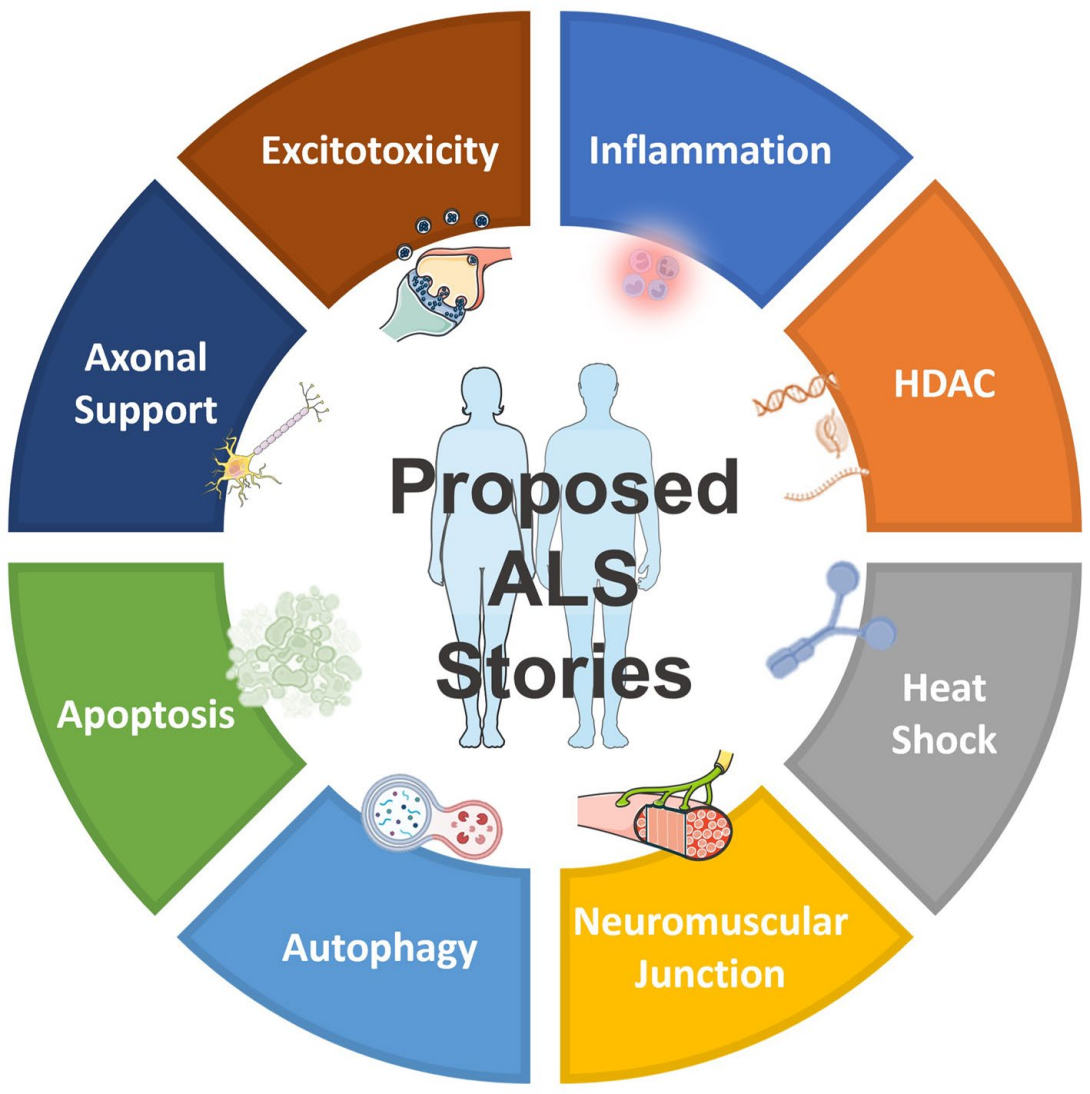
Overview of the proposed target classes for ALS uncovered by NetraAI. Novel genes associated with ALS characteristics can be grouped into 8 target classes: inflammation, epigenetic, heat shock, neuromuscular junction, autophagy, apoptosis, axonal transport, and excitotoxicity.

In addition to the drug targets shown in Table 1, which have already been previously reported and validated, as well as the targets shown in Table 2, which belong to the same gene family as those previously reported (Pun et al., 2022), NetraAI was able to uncover several targets that may shed light into ALS pathophysiology and treatment efforts. Interestingly, these targets can be grouped into a collection or family, called “target classes,” that align to a unique characteristic related to ALS (Figure 2). The target classes discussed here include inflammation, epigenetic, heat shock, neuromuscular junction, autophagy, apoptosis, axonal transport, and excitotoxicity.

These targets are not exhaustive, they represent select target classes that have the potential to play a role in ALS that warrant further investigation. Collectively, these target classes suggest that the simultaneous targeting of several key hallmarks of ALS with combination targeted therapy may have the potential to slow progression, with an enhanced possibility of maintaining and sustaining an improved quality of life (QoL) for certain ALS patients.

### Inflammation target class for ALS

Neuroinflammation is suggested to begin in early ALS pathogenesis, with nervous and peripheral immune systems being impacted (McCauley and Baloh, 2018). Interestingly, we were able to distinguish an inflammation target class involving *TNFα* (Table 3). Given the role of TNFα in immune and inflammatory activity, this is not surprising, considering an innate immune response is characteristic of neurodegenerative diseases like ALS (McCauley and Baloh, 2018). However, the role of TNFα and its receptors TNFR1 and TNFR2 are controversial, with both protective and detrimental effects being reported (Guidotti et al., 2021). Considering that neuroinflammation is a complex and atypical inflammatory process that is meant to protect the central nervous system from injury, in ALS, chronic neuroinflammation can lead to dysregulation that contributes to neurodegeneration. It is now thought that neuroinflammation has dual function, contributing to neuroprotection and possibly leading to neurotoxicity (Tortarolo et al., 2017; Guidotti et al., 2021).

**Table 3:** Inflammation target class in ALS.

Inflammation target class		
Drug target	Function	References
*TNFα* (Tumor Necrosis Factor Alpha)	Mediates immune and inflammatory activity	Tortarolo et al. (2017) and Guidotti et al. (2021)

### Epigenetic target class for ALS

Epigenetic hallmarks have been linked with ALS, specifically with histone deacetylases (HDACs) and their inhibitors, highlighting a potential therapeutic avenue for ALS patients (Klingl et al., 2021). Using the same patient dataset, we also discovered a set of candidate genes that indicate potential HDAC dysregulation and methylation (Table 4). The genes in this target class encode numerous proteins associated with DNA binding and transcription factors, particularly histones and nucleosomes. Although HDAC is a known target for several disease states, including ALS, several HDAC inhibitors currently available have a host of toxic side effects and warrant further investigation to target specific HDACs in specific patient subgroups (Janssen et al., 2010). Collectively, these results highlight the role of epigenetic regulation in ALS pathophysiology.

**Table 4:** Epigenetic target class in ALS.

Epigenetic target class		
Drug target	Function	References
*CHD4* (Chromodomain Helicase DNA Binding Protein 4)	Nucleosome remodeling and deacetylase complex; epigenetic transcriptional repression	CHD4 (n.d.) and Novillo et al. (2021)
*HDAC1, 2, 3, 8* (Histone Deacetylation 1, 2, 3, 8)	Regulators of gene expression and cell differentiation	Janssen et al. (2010)
*REST* (RE1 Silencing Transcription Factor)	Neuronal gene repressor	van Acker et al. (2019)
*HMG20B* (High Mobility Group 20B)	HMG proteins cause changes to chromatin structure by binding to DNA and nucleosomes	GeneCards-HMG20B (n.d.) and Artegiani (2010)
*SAP18* (Sin3A Associated Protein 18)	Histone acetylation; regulation of eukaryotic gene expression	SAP18 (n.d.) and Kumari et al. (2023)
*PHF21A* (PHD Finger Protein 21A)	Histone deacetylase; neuron-specific gene repression	PHF21A (n.d.) and Hakimi et al. (2002)
*KDM1A* (Lysine Demethylase 1A)	Histone demethylase	GeneCards-KDM1A (n.d.) and Casey et al. (2022)
*MTA2* (Metastasis Associated 1 Family Member 2)	Histone deacetylase; nucleosome remodeling	Boulasiki et al. (2023) and MTA3 (n.d.)
*MTA3* (Metastasis Associated 1 Family Member 3)		
*GATAD2B* (GATA Zinc Finger Domain Containing 2B)	Transcriptional repressor	GATAD2B (n.d.) Shieh et al. (2020)
*MBD3* (Methyl-CpG Binding Domain Protein 3)	Nucleosome remodeling and histone deacetylase	Menafra and Stunnenberg (2014)
*RAMP1* (Receptor Activity Modifying Protein 1)	Calcitonin-receptor-like receptor transport	Ringer et al. (2017)

### Heat shock target class for ALS

In ALS, motor neurons have a deficit in the ability to activate the heat shock response (HSR) and do not upregulate the expression of heat shock proteins (Hsps) which are inhibitors of apoptosis and exert an anti-inflammatory response in glia (Apolloni et al., 2019). Here, we were able to uncover a heat shock target class, where the proteins encoded by the genes of interest are primarily associated with protein transport, such as, dynein, actin, and microtubules (Table 5). Evidently, ALS is driven by a collection of genes, with cases being highly heterogeneous; however, protein aggregates in the brain and spinal cord that are positive for *SOD1*, *TDP-43*, or *OPTN* are present in nearly all ALS patients. Under normal physiological conditions, these protein aggregates are prevented and cleared by Hsps, providing further evidence that ALS motor neurons have an impaired ability to induce the HSR (Seminary et al., 2018).

**Table 5:** Heat shock target class in ALS.

Heat shock target class		
Drug targets	Function	References
*HSPBP1* (HSPA (Hsp70) Binding Protein 1)	Ubiquitin protein ligase activity	Jing et al. (2021)
*HSPA5* (Heat Shock Protein Family A (Hsp70) Member 5)	HSP70 chaperone	François-Moutal et al. (2022)
*HSPA9* (Heat Shock Protein Family A (Hsp70) Member 9)		
*ACTR1A* (Actin-Related Protein 1A)	Dynactin complex; ER-Golgi transport; chromosome movement; nuclear positioning; axonogenesis	ACTR1B (n.d.)
*ACTR1B* (Actin Related Protein 1B)		
*DNAJB1* (DnaJ heat shock protein family (Hsp40) member B1)	Molecular chaperone	Dilliott et al. (2022)
*HSPD1* (Heat Shock Protein Family D (Hsp60) Member 1)	Chaperonin family; innate immune signaling	Bross and Fernandez-Guerra (2016)
*DCTN2, 3, 4, 6* (Dynactin Subunit 2, 3, 4, 6)	Dynactin subunits; ER-Golgi transport; chromosome movement; nuclear positioning; axonogenesis	Wang et al. (2018)

### Neuromuscular junction target class for ALS

In the context of ALS, distal axonopathy is a central hypothesis in the early stages of the disease where pathological changes occur at the neuromuscular junction (NMJ). Acetylcholinesterase (AChE) plays a crucial role in nerve-muscle contact, facilitation of neurite outgrowth, and NMJ formation and survival. Interestingly, ALS patients are characterized by abnormal AchE content in plasma, which may reflect neuromuscular disruption (Campanari et al., 2016). Here, we found *HSPG2* to characterize the neuromuscular junction target class (Table 6). Interestingly, a research paper reported on *HSPG2,* among others, as a novel candidate mediator for disease progression. *HSPG2* plays a role in immunological and inflammatory disease, neurological disease, and skeletal and muscular disorders (Morello et al., 2019).

**Table 6:** Neuromuscular junction target class in ALS.

Neuromuscular junction target class		
Drug target	Function	References
*HSPG2* (heparan sulfate proteoglycan 2)	Extracellular matrix and cell-surface crosstalk	Morello et al. (2019)

### Autophagy target class for ALS

Similar to the epigenetic target class, the accumulation of protein aggregates is proposed to disrupt cellular processes that ultimately result in neurodegeneration. Evidently, this protein aggregation in neurons is a hallmark of ALS and may be due to defects in autophagy (Ramesh and Pandey, 2017; Amin et al., 2020). Here, in the autophagy target class, we uncovered several genes implicated in the cellular processes regulating autophagy (Table 7). Autophagy is responsible for maintaining cellular and protein homeostasis in response to nutrient depletion or organelle damage (Ramesh and Pandey, 2017). However, it is still unknown whether activation or inhibition of autophagy would be most effective in the treatment of ALS (Nguyen et al., 2019). Interestingly, *SOD1* is a frequent ALS mutation and it is expected that aggregation of mutant *SOD1* (mSOD1) is a crucial event in ALS pathogenesis, and dysregulation of autophagy has been linked to SOD1 aggregates in motor neurons (Nguyen et al., 2019). This highlights the need to study further and identify therapeutic agents that target the clearance of these protein aggregates.

**Table 7:** Autophagy target class in ALS.

Autophagy target class		
Drug target	Function	References
*MAP1LC3A* (Microtubule-associated protein 1 light chain 3 alpha)	Autophagosome biogenesis and transport	Bonam et al. (2020)
*ATG3* (autophagy-related 3)	Autophagy regulation	Ramesh and Pandey (2017)
*GABARAP* (GABA Type A Receptor-Associated Protein)	Autophagosome movement; protein aggregation inhibition; cytoskeleton interaction	Brennan et al. (2022)
*ATG4C* (autophagy-related 4C cysteine peptidase)	LC3 conjugation system	van Beek et al. (2018)

### Apoptosis target class for ALS

In ALS, there is evidence of apoptosis through DNA fragmentation, caspase-9 activation, BAX overexpression, and reduced Bcl-2 expression (Erekat, 2022). Interestingly, m*SOD1* induces apoptosis via cytochrome c release and Bcl-2 degradation (Erekat, 2022). As a result, treatments targeting apoptosis can be helpful in rescuing neurons from cell death. In the apoptosis target class, several caspases as well as apoptotic mediators were identified (Table 8). Of note is that of the caspases identified, based on their mechanism of action and their position in the apoptotic signaling pathways, apoptotic caspases can be initiatory caspases (caspase 2, 8, 9, and 10) and executioner or effector caspases (caspases 3, 6, and 7) (Erekat, 2022). As a result, similar to autophagy, whether promoting or inhibiting critical caspases involved in apoptosis presents as a therapeutic approach for ALS patients.

**Table 8:** Apoptosis target class in ALS.

Apoptosis target class		
Drug target	Function	References
*CASP7, 8, 9, 3* (Caspase 7, 8, 9, 3)	Execution phase of apoptosis	Pasinelli and Brown (2006)
*FADD* (Fas Associated Via Death Domain)	Death signaling	Raoul et al. (2002)
*FAS* (Fas cell surface death receptor)	Death signaling; caspase cascade	Raoul et al. (2002)
*APPL1* (Adaptor Protein, Phosphotyrosine Interacting with PH Domain And Leucine Zipper 1)	Metabolic and inflammatory response regulator	Hsu et al. (2018)
*DIABLO* (Diablo IAP-Binding Mitochondrial Protein)	Caspase activation	Benn and Woolf (2004)

### Axonal transport target class for ALS

Neurons have long axonal projections that rely on cytoskeletal integrity to maintain axonal stability, transport, and signaling (Theunissen et al., 2021). In ALS there is selective, early degeneration of motor neurons in the brain and spinal cord. Related to this, we identified a target class characterized by several genes that play a role in microtubule cytoskeletal organization (Table 9). Disrupted transport mechanisms can affect mitochondrial metabolism and degeneration, protein degradation, and RNA transport, collectively resulting in motor neuron death (Le Gall et al., 2020). Furthermore, within this target class, we identified *TARDBP* and *RPA1* which have been implicated in ER-Golgi transport dysfunction that is associated with ALS (Soo et al., 2015). It is important to note that in this target class, we identify two HDACs, and conversely, in the heat shock target class, we identified dynactin. This observation demonstrates that ALS pathophysiology is characterized by overlapping systems and is heterogeneous (Le Gall et al., 2020). It should be emphasized that even though these gene candidates are organized under specific categories, the manifestation of the disorder, and the potential treatments, all depend on the fact that the corresponding proteins, and higher-order systems, interact with each other. Hence, these findings should not be considered isolated processes but parts of an emergent system.

**Table 9:** Axonal transport target class in ALS.

Axonal transport target class		
Drug target	Function	References
*GSK3B* (Glycogen Synthase Kinase 3 beta)	Cell signaling regulation	Choi et al. (2020)
*CCT2, 3, 4, 5, 6, 7, 8* (Chaperonin Containing TCP1 Subunit 2, 3, 4, 5, 6, 7, 8)	Ubiquitin protein ligase binding; unfolded protein binding	Bernardini et al. (2013) and Kim et al. (2017)
*TUBA1C* (Tubulin Alpha 1c)	Microtubule cytoskeleton organization	Buscaglia et al. (2020)
*LRRC49* (Leucine Rich Repeat Containing 49)	Protein metabolism; actin and tubulin folding	GeneCards-LRRC49 (n.d.)
*TCP1* (T-Complex 1)	Protein folding	Khorkova and Wahlestedt (2017)
*TUBB1* (Tubulin Beta 1 Class VI)	Neurogenesis; axon guidance and maintenance	Morello and Cavallaro (2015)
*TUBB2A* (Tubulin Beta 2A Class IIa)		
*HDAC6* (Histone Deacetylase 6)	Deacetylation; epigenetic repression and transcriptional regulation	Fischer et al. (2010) and Taes et al. (2013)
*HDAC7* (Histone Deacetylase 7)		
*TARDBP* (TAR DNA binding protein)	RNA-binding protein involved in RNA biogenesis and processing; maintaining mitochondrial homeostasis	Todd et al. (2011)
*RPA1* (replication protein A1)	Stabilizes single-stranded DNA intermediates; DNA replication and cellular response to DNA damage	Haring et al. (2008)

### Excitotoxicity target class for ALS

Finally, we extracted a collection of genes implicated in excitotoxicity (Table 10). Excitotoxicity is a phenomenon that describes the toxic actions of excitatory neurotransmitters where prolonged activation starts a cascade of neurotoxicity that ultimately leads to the loss of neuronal function and cell death (Armada-Moreira et al., 2020). Importantly, excitotoxicity can both contribute to as well as be a result of other deregulations, including mitochondrial dysfunction, neuronal damage, and oxidative stress (de Marco et al., 2022). Similar to other target classes, there is evidence that dysregulation of mitochondrial calcium handling plays a role in excitotoxicity (Verma et al., 2022).

**Table 10:** Excitotoxicity target class in ALS.

Excitotoxicity target class		
Drug target	Function	References
*GRIA3* (glutamate ionotropic receptor AMPA type subunit 3)	Glutamate receptor	Comabella et al. (2009)
*AKAP5* (A-kinase anchoring protein 5)	Synaptic plasticity and memory	Comabella et al. (2009)
*GRIN1* (Glutamate Ionotropic Receptor NMDA Type Subunit 1)	Glutamate NMDA receptor; mediator of excitotoxicity	Young et al. (2017)
*CAMK2A* (calcium/calmodulin-dependent protein kinase II alpha)	Calmodulin-dependent activity; long-term potentiation; learning	Honda et al. (2014)
*ACTN2* (Actinin Alpha 2)	Cytoskeleton protein scaffold	Savarese et al. (2020)

### Drug target confidence evaluation

Utilizing an Open Source bioinformatics tool, TargetMine, we evaluated the confidence in the drug targets in the manuscript thus far (Chen et al., 2022). Adding our target genes to TargetMine we were provided with 11 overarching pathway categories, each with varying levels of confidence (Table 11). Of the 86 targets, 36 were associated with pathways of neurodegeneration including ALS, with a high level of confidence (3.4×10^-16^). Interestingly, 30 targets were also associated with SARS-CoV infection and interferon signaling, 35 targets were associated with RHO GTPase effectors, nuclear receptor signaling, chromatin modifying enzymes and viral carcinogenesis, 61 targets were associated with nervous system development, and 43 targets were associated with homeostasis and the neuronal system, all with high levels of confidence. All of the targets identified to be associated with cell cycle, transcriptional dysregulation in cancer, organelle biogenesis and maintenance, carboxyterminal post-translational modification of tubulin, bacterial infection pathways, and autophagy, which, despite having a lower confidence level, highlight that ALS may be a complex disorder. However, an alternative explanation is that there is a historical bias toward favored pathways and that genes are inherently promiscuous, making our molecular machinery highly connected. The output of the TargetMine software is included as a Supplementary file, which includes one table outlining the statistical significance of the pathways enriched for and the other with the pathways and genes themselves.

**Table 11:** Pathway involvement and confidence of NetraMark identified targets.

Associated pathways	Confidence level
Pathways of Neurodegeneration – Multiple Diseases: Alzheimer’s Disease | Amyotrophic Lateral Sclerosis	
*ACTR1A, ACTR1B, CAMK2A, CASP3, CASP7, CASP8, CASP9, DCTN2, ERN1, FADD, FAS, GRIA3, GRIN1, GSK3B, HDAC1, HDAC2, HDAC6, HSPA5, KDM1A, MAP1LC3A, MAP3K5, MAPK1, NOS1, PPP3CB, PTK2, REST, RPS6KA1, RPS6KA2, SLC25A4, SLC25A5, SLC25A6, TARDBP, TUBA1C, TUBB1, TUBB2A, VCP*	3.4×10^-16^
SARS-CoV Infection | Interferon Signaling	
*CAMK2A, CHD4, DNAJB1, DNMT3A, GATAD2B, GSK3B, HDAC1, HDAC2, HDAC3, HDAC6, HDAC7, HDAC8, HMG20B, HSPA5, HSPA9, HSPG2, KDM1A, MAPK1, MBD3, MTA2, MTA3, NR3C2, PHF21A, PPIA, RARA, REST, RPA1, SAP18, TUBB2A, VCP*	6.55×10^-13^
RHO GTPase Effectors | Signaling by Nuclear Receptors | Chromatin Modifying Enzymes | Viral Carcinogenesis	
*CASP3, CASP8, CHD4, DNMT3A, FADD, GATAD2B, GRIN1, GSK3B, HDAC1, HDAC2, HDAC3, HDAC6, HDAC7, HDAC8, HMG20B, HSPG2, KDM1A, MAP3K5, MAPK1, MBD3, MTA2, MTA3, PHF21A, PTK2, RARA, REST, RPA1, RPS6KA1, RPS6KA2, SAP18, SLC25A4, SLC25A5, SLC25A6, TUBB2A, VCP*	2×10^-10^
Nervous System Development | Pathways in Cancer | Herpes Simplex Virus 1 Infection | Signaling by Receptor Tyrosine Kinases | Signaling by Interleukins | Diseases of Signal Transduction by Growth Factor Receptors and Second Messengers | PI3K-Akt Signaling Pathway | Human Papillomavirus Infection | MicroRNAs in Cancer	
*ACTN2, ACTR1A, ACTR1B, AKAP5, APPL1, ATG3, ATG4C, CAMK2A, CASP3, CASP7, CASP8, CASP9, CHD4, DCTN2, DIABLO, DNAJB1, DNMT3A, ERN1, FADD, FAS, GABARAP, GATAD2B, GRIA3, GRIN1, GSK3B, HDAC1, HDAC2, HDAC3, HDAC6, HDAC7, HDAC8, HSPA5, HSPA9, HSPD1, HSPG2, KDM1A, MAP1LC3A, MAP3K5, MAPK1, MBD3, MTA2, MTA3, NOS1, NR3C2, P2RY12, PPIA, PPP3CB, PTK2, PTPRC, RARA, REST, RPS6KA1, RPS6KA2, SLC25A4, SLC25A5, SLC25A6, TCP1, TUBA1C, TUBB1, TUBB2A, VCP*	2.14 × 10^-9^
Homeostasis | GPCR Ligand Binding | Neuronal System	
*ACTN2, AKAP5, CAMK2A, CASP3, CCT2, CCT3, CCT4, CCT5, CCT6A, CCT7, CCT8, DCTN2, GABARAP, GRIA3, GRIN1, GSK3B, HDAC1, HDAC2, HDAC3, HMG20B, HSPA5, KCNB1, KCNV2, KDM1A, MAP1LC3A, MAP3K5, MAPK1, NOS1, P2RY12, PHF21A, PPIA, PPP3CB, PTK2, RAMP1, RPS6KA1, RPS6KA2, SLC25A4, SLC25A5, SLC25A6, TCP1, TUBA1C, TUBB1, TUBB2A*	2.42×10^-9^
Cell Cycle | Mitotic | Class I MHC Mediated Antigen Processing and Presentation | Signaling by WNT | Deubiquitination	
ACTR1A, APPL1, CAMK2A, CASP3, CASP7, CASP8, CASP9, DCTN2, DIABLO, FADD, FAS, GSK3B, HDAC1, HDAC2, HDAC3, HDAC6, HDAC7, HDAC8, HSPA5, MAPK1, PPIA, PPP3CB, PTK2, PTPRC, RPA1, SLC25A4, SLC25A5, SLC25A6, TUBA1C, TUBB1, TUBB2A, VCP	0.000306459
Transcriptional Misregulation in Cancer | Cell Cycle | Direct p53 Effectors | C-MYB Transcription Factor Network | Glucocorticoid Receptor Regulatory Network	
*AKAP5, CASP3, CASP7, CASP8, CASP9, DNMT3A, FAS, GRIA3, GSK3B, HDAC1, HDAC2, HDAC3, HDAC7, HDAC8, HSPD1, MAPK1, PPP3CB, PTK2, RARA, SAP18*	0.00092631
Organelle Biogenesis and Maintenance	
*ACTR1A, CCT2, CCT3, CCT4, CCT5, CCT8, DCTN2, HDAC3, HDAC6, HSPA9, RARA, TCP1*	0.004850972
Carboxyterminal Post-Translational Modifications of Tubulin	
*LRRC49, TUBA1C, TUBB1, TUBB2A*	0.008692446
Bacterial Infection Pathways | Transcriptional Regulation by MECP2	
*CAMK2A, HDAC1, HDAC2, HDAC3, MAPK1, REST, VCP*	0.009967429
Autophagy	
*ATG3, ATG4C, GABARAP, HDAC6, MAP1LC3A, VCP*	0.021986433

### Identification of drivers of a subpopulation of limb and bulbar onset ALS patients

**Figure 3:**
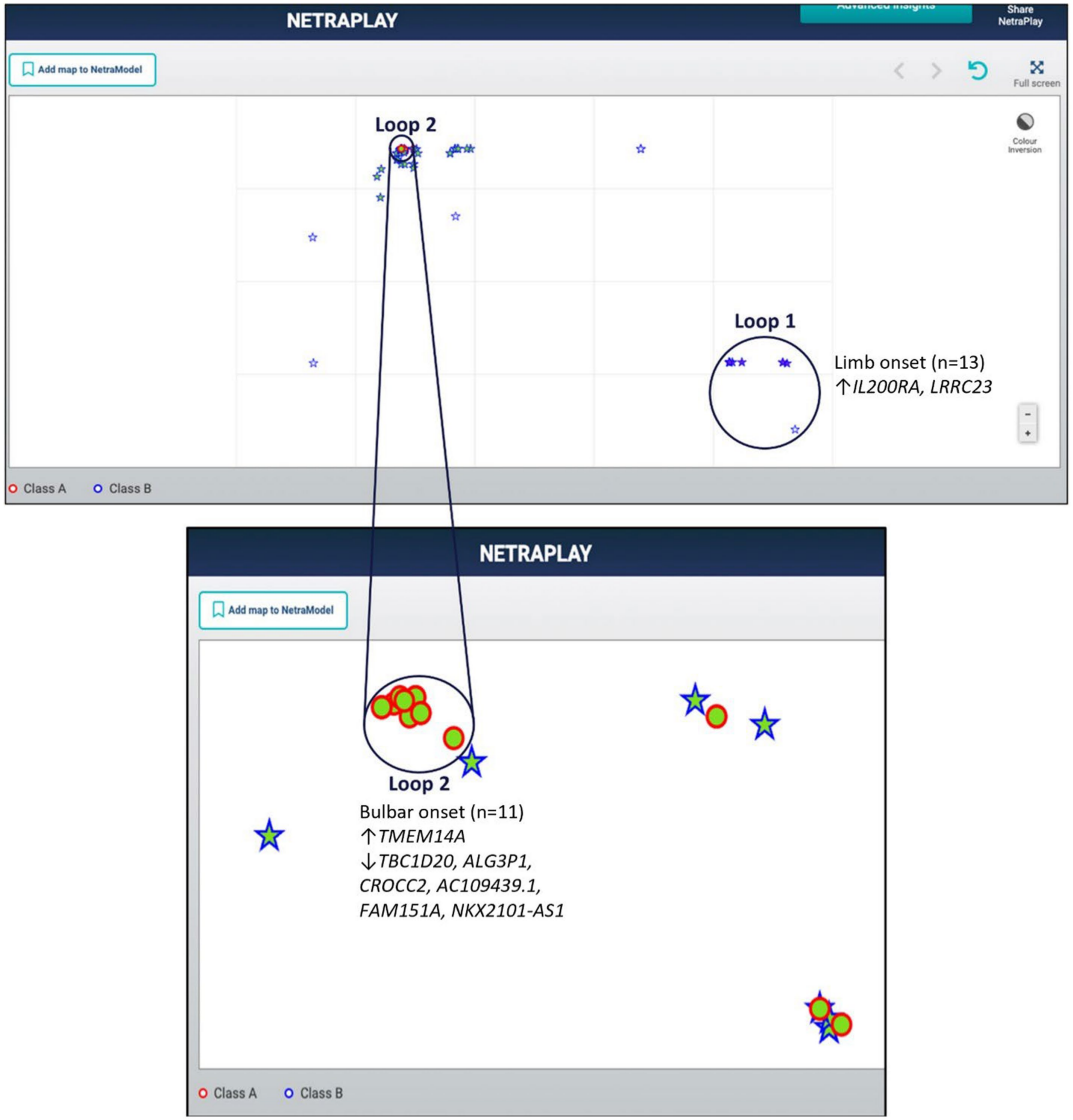
Map of limb and bulbar ALS patients. Class A (red circles) indicate bulbar-initiated samples and Class B (blue stars) indicate limb-initiated samples. Loop 1 corresponds to a subpopulation of limb onset ALS patients. Loop 2 corresponds to a subpopulation of bulbar onset ALS patients. Loop 2 consists of a hidden group of 11 bulbar initiated samples and Loop1 consists of 13 limb associated samples. Note that in this representation the samples are so close to each other that some of the samples within the loops are obfuscated.

**Figure 4:**
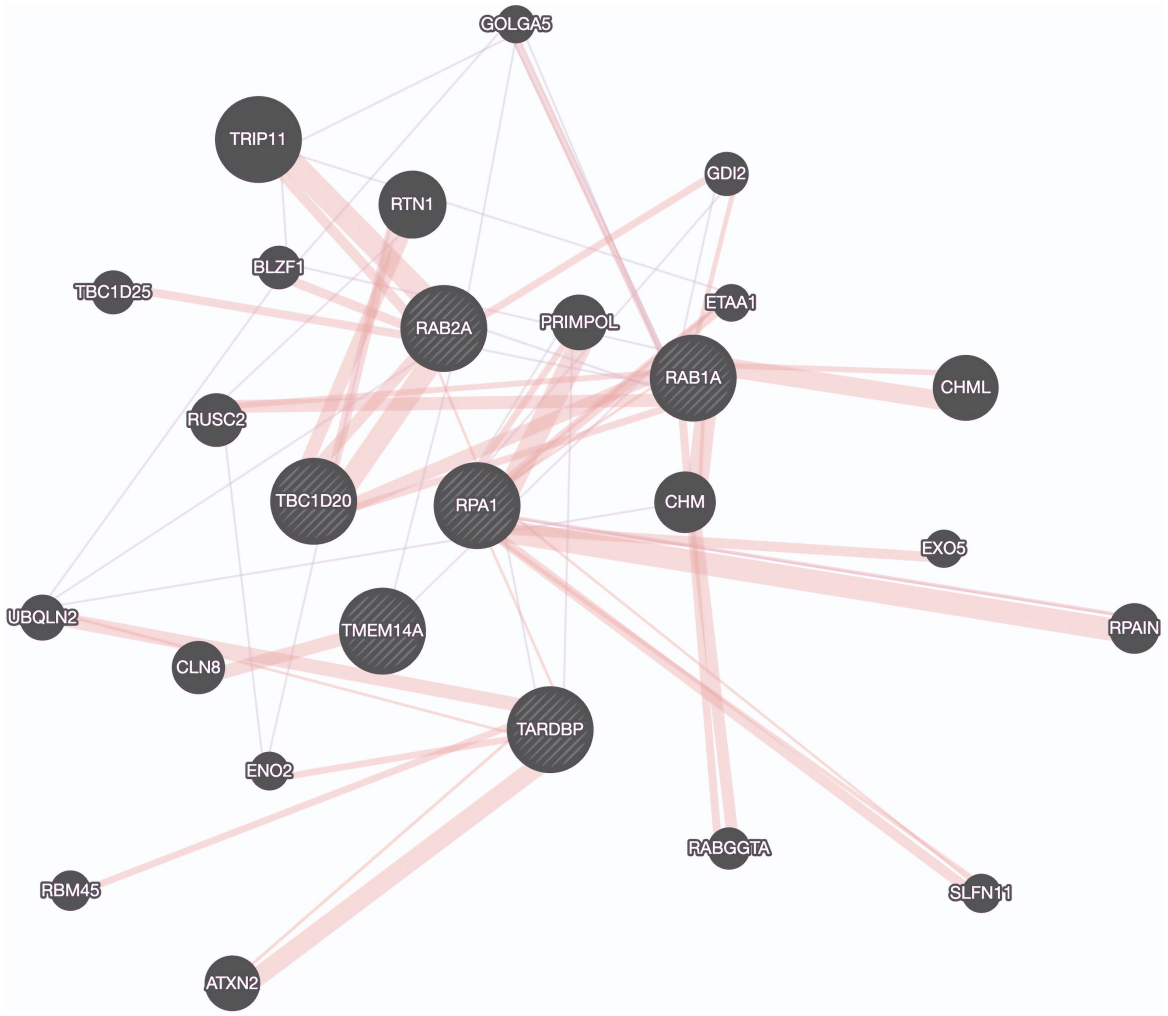
Protein Interaction Map Revealing connections to TDP-43. Protein interaction network derived by genes found in a potentially aggressive subtype of bulbar onset ALS driven by TBC1D20, TMEM14A, RAB1A, RAB2A, TDP-43 (TARDBP), and RPA1. Purple edges represent co-expression and pink lines represent physical interactions. Created using GeneMania.

Utilizing a dataset consisting of 31 bulbar onset and 85 limb onset ALS patients, we identified distinct subpopulations, each defined by a specific set of driving genes (Figure 3). A subpopulation of 13 limb onset ALS patients was identified to be characterized by an elevated expression of *IL200RA* and *LRRC23* (Loop 1). Even further, we identified a distinct subpopulation of 11 bulbar onset ALS patients (Loop 2) that was characterized by a decreased expression of *TBC1D20*, *ALG3P1*, *CROCC2*, *AC109439.1*, *FAM151A*, and *NKX2101-AS1*, and an elevated expression of *TMEM14A.* The remaining limb onset patients, which comprised the majority of the dataset, were characterized by expression patterns opposite to the bulbar subpopulation – specifically increased expression of *TBC1D20*, *ALG3P1*, *CROCC2*, *AC109439.1*, *FAM151A*, and *NKX2101-AS1*, and decreased expression of *TMEM14A.* These findings indicate that specific genetic factors may accurately delineate novel subtypes of bulbar and limb-initiated ALS. Unraveling these subpopulations has significant implications for clinical trials, as it can unveil alternative etiological subtypes that might respond more favorably to particular therapeutic interventions. A gene interaction network constructed of *TMEM14A* and *FAM151A,* revealed nearest neighbor connections to *RAB1, RAB2*, and *TDP-43 (TARDBP in the gene interaction figure),* suggesting the identification of a more aggressive ALS subpopulation within the bulbar onset patients (Figure 4).

**Figure 5:**
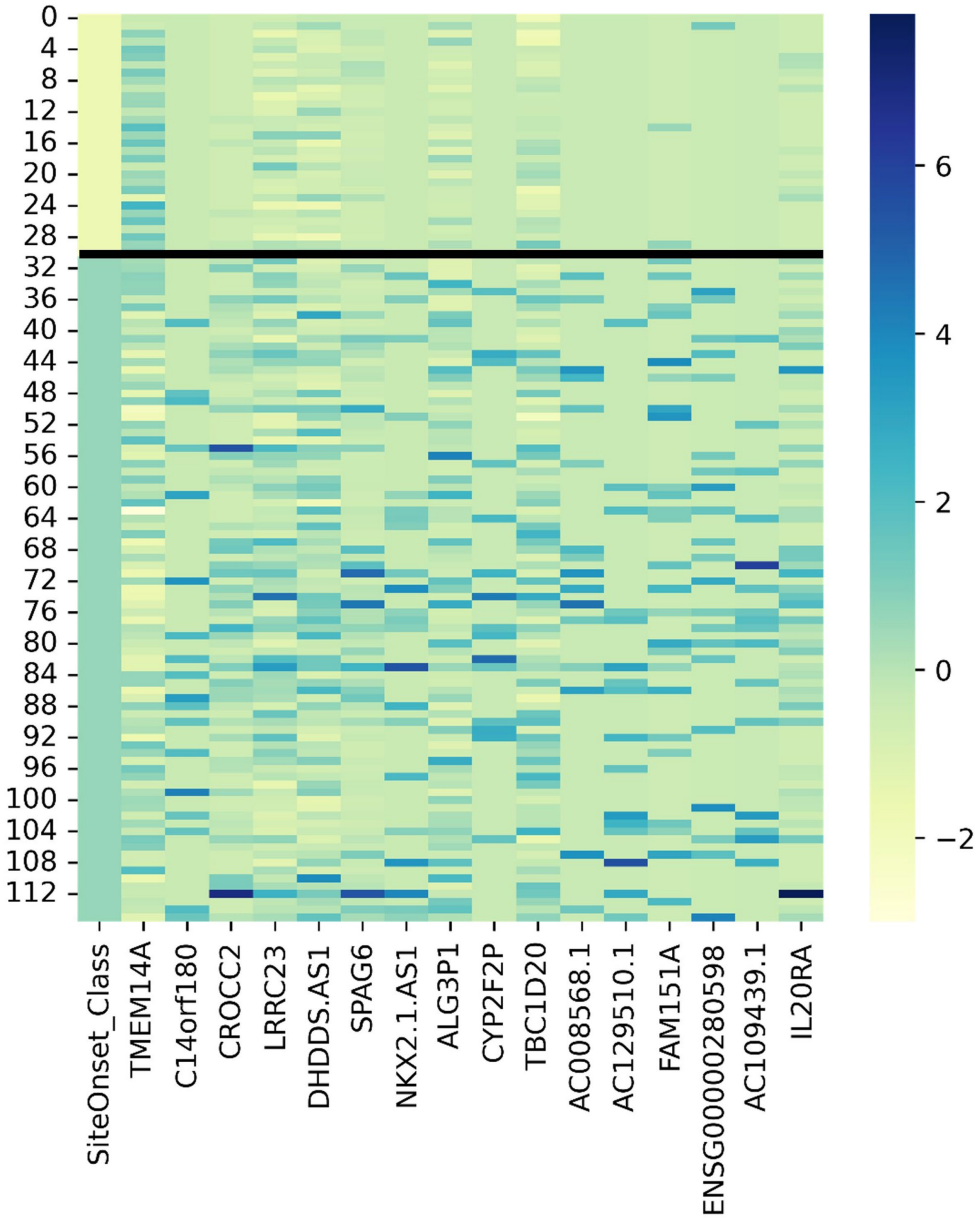
Gene expression heatmap for discovered genes driving certain subpopulations of bulbar and limb initiation samples. Note the first column is the label where the first 31 samples are from patients with bulbar initiation and the remaining 85 samples are from patients with limb initiation.

We adopted z-score normalization prior to generating the heatmap (Figure 5) facilitated by the Seaborn Python library (Waskom, 2021). It is evident that certain genes distinctly differentiate the samples across respective classes. However, a limitation of this visual representation is the inability to distinctly highlight the subpopulations present within the heterogeneous sample group. This distinction emerges prominently through ML applications, where synergistic effects arise from integrating multiple variables concurrently. Nonetheless, the distinctiveness of several genes can be ascertained by contrasting the intensities above and below the demarcating black bar in Figure 3. The heatmap corroborates the highlighted trends explained in Figure 3, specifically that *TMEM14A* is upregulated for bulbar-initiated samples, while *TBC1D20*, *ALG3P1*, *CROCC2*, *AC109439.1*, *FAM151A*, *NKX2.1.AS1* are all downregulated. *IL20RA* and *LRCC23* are upregulated for limb-initiated samples, especially for the 13 samples represented in Loop 1 of Figure 3.

We employed two classifiers, namely Random Forest and Gradient Boosted Trees, to assess their performance using a leave-out cross-validation approach. The Gradient Boosted Trees exhibited an accuracy of 70.7% in 10-fold cross-validation and 73.9% in 5-fold cross-validation, while the Random Forest classifier performed slightly better with accuracies of 74.1 and 75% in the respective cross-validation schemes. These results suggest the presence of discernible patterns within the data.

To validate the robustness of these subtype discoveries alongside the previously mentioned driving transcriptomic factors, we constructed a new dataset comprising only these relevant variables and re-evaluated the classifiers using leave-out cross-validation. Notably, the use of this reduced dataset led to enhanced model accuracy. For instance, complex models like Random Forest yielded accuracies exceeding 80% in both the 10-fold and 5-fold cross-validation iterations. Most notably, simpler models like logistic regression, which initially exhibited poor performance with an accuracy of approximately 65%, now generated stable models with an impressive accuracy of approximately 84% for both 10-fold and 5-fold cross-validations.

These findings highlight the utility of our approach in identifying subpopulations and driving transcriptomic factors, which can be further scrutinized through bioinformatics analyses. The improved accuracy of the models underscores the importance of considering these factors when characterizing ALS subtypes and devising tailored therapeutic strategies.

These targets were discovered after allowing ML to generate hypotheses about important genetic variables using the knowledge of protein–protein interactions and co-expression to extend our search. Protein interaction networks represent a rich source of data for understanding complex biological systems and deriving potential drug targets. These networks represent nodes and their interactions as edges, forming a complex graph that can be analyzed using various network analysis techniques.

## Discussion

ALS is the most common motor neuron disease in adults and the third most common neurodegenerative disease; yet this debilitating disease has no cure due to gaps in our understanding of disease etiology and treatments focused on improving symptoms (Logroscino et al., 2018). In the spirit of Open Innovation, the EndALS Challenge was designed to connect the data science and AI community with neuroscientists to bridge the gap associated with ALS diagnosis and drug discovery (Armada-Moreira et al., 2020). EndALS was developed by not-for-profit organizations focused on helping ALS patients (EverythingALS and Answer ALS) in collaboration with Roche’s AI Center of Excellence, “AI with Roche” (a.k.a.aiR), Canadian public and private organizations (ALS Society of Canada, Ontario Brain Institute (OBI), and NetraMark Corp.), and administered by the data science and ML community platform Kaggle. The main mission has been to push the boundaries of knowledge in ALS biology to help with the diagnosis and therapeutic strategies for ALS patients (Armada-Moreira et al., 2020). This report was aimed at being a follow-up of the PandaOmics paper that focused on the identification therpauetic targets for ALS using an AI-enabled biological target discovery platform (Pun et al., 2022). We reported on several genes that have been previously reported to be implicated in ALS Table 1, genes that belong to the same family as those previously reported Table 2, as well as genes that belong to the same protein family as those previously reported (Table 2), as well as 8 target classes that correspond to key characteristics of the disease: inflammation, epigenetic, heat shock, neuromuscular junction, autophagy, apoptosis, axonal transport, and excitotoxicity (Figure 2). The results presented in Tables 1, 2 are reported as they validate genes previously reported to be implicated in ALS as well as corroborate the results obtained using NetraAI (Pun et al., 2022). Even further, we identified a set of genetic drivers that differentiate between subpopulations of limb and bulbar onset ALS patients. Figure 3 was generated using a proprietary visualization technology and was previously employed to explore patient relationships in Alzheimer’s disease, bipolar disorder, and lung cancer (Qorri et al., 2020; Choi et al., 2021; Cook et al., 2023). This technology, known as NetraPlay, complements standard ML pipelines, including those described in this paper and the cited works. It enables the discovery of hidden relationships from multimodal data, ensuring complete explainability without complex latent variables, as detailed in the referenced papers. To ensure reproducibility, interested readers may request access to a secure instance of NetraPlay by contacting the first author. Furthermore, by leveraging the insights presented here, readers can verify the characterization of a subset of samples from the bulbar vs. limb data based on a set of transcriptomic markers.

In this way, the 8 target classes extracted using NetraAI highlight genetic drivers that are associated with subgroups of patients that can be useful in matching patients to therapy as well as for drug discovery in ALS. This is further supported by the stratification we identified in subpopulations of ALS patients based on disease onset. Thus, a personalized medicine approach can be made possible to pair patients to treatment(s) that address the target classes applicable to each patient through focused screening. Further, clinical trials in this space can benefit by understanding which patient subpopulations are best aligned with the mechanism of action of their drug, thereby improving drug response signal.

Notably, the target classes we uncovered and the broad ALS characteristics they correspond to are not novel on their own, but rather the combination of genes driving each target class are novel (Figure 2). Even though each target class has its own overarching characteristic, we noticed that some target classes also included genetic drivers related to other target classes. For example, in the heat shock target class (Table 5), there was the presence of dynactin, and in the axonal transport target class (Table 9), there were two HDAC genes. These results further support the claim that ALS is a multisystem disorder.

Further evidence of the complexity of ALS is highlighted in Table 11, where 11 primary pathway categories were identified that the targeted genes reported in this paper play a role in, with varying degrees of confidence. Although many targets were identified to belong to pathways of neurodegeneration for diseases including ALS, the other pathways raised interesting points of discussion. Of particular interest was the second category, namely SARS-CoV infection and interferon signaling. There have been reports linking interferon signaling to ALS, suggesting an early interaction between motor neurons and astrocytes during the pathological changes that take place in ALS (Wang et al., 2011). Additionally, a recent study focusing on the role of type I interferon response highlights that the role of interferon signaling in the absence of bacterial or viral infection can be detrimental as noted in several neurological disorders, including ALS (Vitner et al., 2016). These reports, among others highlight the importance of interferon signaling in ALS that warrants continued investigation, as well as explains why viral infection reappeared within several of the category pathways.

With respect to the stratification based on disease onset, the gene network connections to *RAB1*, *RAB2*, and *TDP-43* which are known for their roles in intracellular transport, suggest that intracellular transport dysfunction may be a hallmark of bulbar onset ALS (Burk and Pasterkamp, 2019). This finding underscores the significance of *TDP-43* in ALS pathophysiology through a physically interacting protein encoded by *RPA1*. Previous studies have implicated the roles of *RAB1* and *RAB2* in disrupted vesicle trafficking in ALS, but not for this specific subpopulation (Parakh et al., 2018). This finding might indicate a more aggressive form of the disease and provides additional evidence pointing to the significant role of *TDP-43* in ALS. Further, this highlights the role of *RPA1* as a biomarker for this subpopulation.

In this report, we set out to present a set of targets associated with the complex and heterogeneous disease of ALS. While some targets reported here have been linked and associated with ALS previously, validating the impact of the novel ML methods employed by NetraAI, others did not initially have a direct link to ALS or were not supported with high confidence levels. Since we were able to accurately and efficiently identify previously reported targets, we can with some level of confidence claim that these novel targets are playing a role in the manifestation of ALS pathophysiology. However, a limitation of this report lies in that it is an in-silico exploration of data. Despite using techniques that have been validated in other studies, the outcomes of this report are hypotheses that can be used as a framework for future studies in the nature of the disease as well as for drug discovery and development.

The findings presented in this report highlight the magnitude of meaningful results that can be obtained from the intersection of AI/ML with scientists, biologists, and the public, implicit to the concept of Open Science. Physicians and medical scientists spend decades becoming content experts in the details of a disease, the experience of the patient population, and the etiological factors that influence prognosis and the course of the disease. Currently, most groups utilizing ML are siloed into computer science and medical or research teams, where the groups struggle to communicate and collaborate. Fortunately, there are now tools that provide a platform for medical scientists to be involved in the model selection process, bridging the enormous gap that currently exists between these different areas of expertise.

Open Science tools can potentially capture the lived experience of clinicians and integrate this into AI/ML analyses. Our approach was to utilize ML algorithms to generate hypotheses surrounding the pathophysiology of ALS. By fusing this analysis with other systems biology tools, the target lists extend to genetic, co-expression, and protein interaction networks. As a result, these augmented intelligence tools can generate three kinds of hypotheses:

- What are groups of patients most closely related to each other?
- What genetic factors explain this grouping?
- What proteins can be potential drug targets?

In turn, these hypotheses can be tested for statistical significance and, more importantly, can be evaluated for clinical significance by physicians and biologists for context and biological plausibility.

In general, most enterprise data is unstructured, and this includes text, speech, imaging, and PDF files, all related to clinical encounters, with volumes of data rapidly growing with the adoption of electronic health records. ML in combination with data analysis can improve drug development, particularly in identifying accurate biomarkers and developing predictive models (Vamathevan et al., 2019). However, the main challenge with working with patient populations is the lack of large datasets, where there are insufficient numbers of samples despite having up to tens of thousands of variables that ML can learn from. Thus, there is an increased need to develop techniques amenable to small datasets, such as the methods utilized for the discovery of the targets reported in this paper. Furthermore, methods that create artificial data representations of the patient population are also being considered (Silva, 2019). Methods like this attempt to embed the data into a geometric space so that learning becomes augmented by elucidating structures within the data (Qi and Luo, 2022). Other methods involve generating more data, assuming the original dataset is of high enough quality. This utilizes a type of ML that is referred to collectively as generative ML, and of recent interest are generative adversarial networks (Ashrapov, n.d.). These ML methods learn from the available data and then create artificial datasets that can then be used to create predictive models.

The approach used to generate the list of potential drug targets for ALS relied on the idea that statistics is a very powerful tool to assign some level of confidence to hypotheses. This means that if we had a system that could generate hypotheses, then we could use statistics and human expertise to evaluate them. In the case used here, these hypotheses are collections of samples and a collection of genes. These insights can be evaluated through statistical significance testing and simultaneously reviewed for biological plausibility. Hypotheses that survive this dual scrutiny can then be pushed forward for more research. Importantly, we recognize that small datasets often do not capture the heterogeneity involved for complex disorders; however, it is very possible that part of the distribution is captured, and novel insights gleaned. Future work should be focused on experimental validation of novel potential targets described here, to confirm their functional relevance in ALS pathophysiology. Furthermore, the interactions between the target classes can assist in gaining a more comprehensive understanding of the multifaceted nature of ALS.

## Conclusion

In the spirit of Open Science, the results highlighted in this paper emphasize the impact that advancements in ML approaches in collaboration with scientific and medical researchers hold on the potential to revolutionize drug discovery and development. By using a small ALS dataset and a unique combination of ML methods, we have not only validated previously reported drug targets in ALS but also uncovered critical insights into ALS subpopulations. Our findings encompass 8 target classes of genes that relate to ALS pathophysiology that inform on its etiology and represent novel drug targets, as well as identify a unique, potentially more aggressive subpopulation of bulbar onset ALS patients that are characterized by a distinct set of genetic drivers. This systematic view offers the promise of simultaneously targeting multiple aspects of ALS to mitigate disease progression and enhance the QoL of patients. Furthermore, our identification of subpopulations based on disease onset paves the way for personalized treatments, tailored to individual needs, highlighting the importance for open data efforts in rare diseases.

Open Science is being increasingly adopted, with national and global movements to bridge the knowledge gap that currently exists between AI/ML, and scientific and medical researchers. In line with these movements, Open Science has enabled us to derive meaningful insights into the etiology of ALS. This highlights the global benefit that this approach can have. However, as this is an evolving framework, greater adoption, caution, and deep expertise is required of the researchers before navigating this landscape. The work further highlights the importance of ML methods that can handle smaller sample sizes through the generation of hypotheses, as this allowed for the extraction of targets that required much larger datasets to reveal through more data expensive methods.

## Data availability statement

Publicly available datasets were analyzed in this study. This data can be found here: The data used in the preparation of this article were obtained from the Answer ALS Data Portal (AALS-01184). For up-to-date information on the study and access to the data please visit https://www.answerals.org/.

## Ethics statement

Ethical review and approval was not required for the study on human participants in accordance with the local legislation and institutional requirements. Written informed consent from the patients/participants was not required to participate in this study in accordance with the national legislation and the institutional requirements.

## Author contributions

FS and JG contributed to conception of the study. JG was responsible for the design of the study and research. RB, FS, BQ, and MC wrote sections of the manuscript. DC and LP reviewed, edited and provided medical expertise. All authors contributed to manuscript revision, read, and approved the submitted version.
